# Enhanced Implications on Turbidity Removal from Natural Stone Wastewater by Binary Mixtures

**DOI:** 10.1021/acsomega.4c01448

**Published:** 2024-06-22

**Authors:** Savas Ozun

**Affiliations:** Department of Mining Engineering, Süleyman Demirel University, Isparta 32260, Turkey

## Abstract

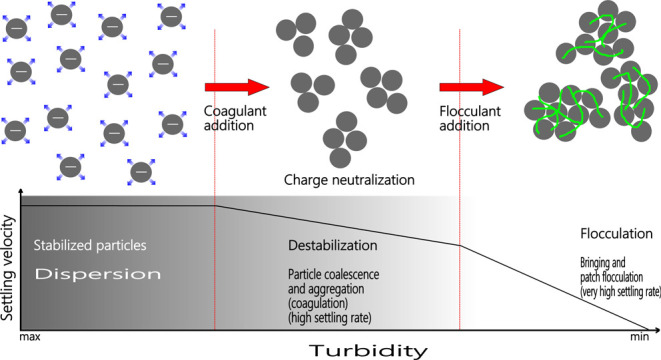

The settling rate of the mineral fines in an aqueous solution changes depending on the charges they carry. Mineral fines with similar high-magnitude surface charges repel each other and prevent them from settling rapidly. In contrast, fines with no/low-magnitude surface charges can coalesce and agglomerate with the others and settle rapidly due to the increasing mass. This can lower the coagulant or flocculant use and speed up turbidity removal. Thus, considering this fact, the experimental tests in this study were performed below the neutral pH environment (pH 2–6) to determine the effectiveness of the coagulant and flocculant mixtures and compare the results with their single use. The turbidity removal tests were applied using different valence coagulants and flocculants with different charge mechanisms. According to the results with their single use, the best results were obtained using FeCl_3_ (80 mg/L) at pH 4 with a turbidity removal efficiency of ≤98% and a nonionic flocculant at pH 2 with a turbidity removal efficiency ≥99% (0.50 mg/L). When they were used as binary mixtures, the lowest turbidity values were obtained with FeSO_4_/nonionic flocculant mixtures at pH 4 (≤98%) and with FeCl_3_/anionic flocculant mixtures at pH 2 (≥99%).

## 1 Introduction

Large amounts of water are used in the mining and cutting/processing of natural stone (granite, marble, basalt, etc.) to reduce the risk of frictional ignitions and extend the lifespan of cutting and sizing units and diamond wires. Another primary purpose of water is to transport the fine particles formed by sizing natural stone blocks to tailing pond. About 25–40% of the products are mixed in the water during these operations, forming wastewater-containing solids to 2–10% of the products by weight.^1−5^

In mining and natural stone-cutting/processing plants, wastewater mainly contains fine mineral particles and water. Although many factors like size, shape, density, and surface charge of the particles impact the pace at which they settle during sedimentation, the substantial density difference between mineral fines and liquids (mostly water) in wastewater makes the sedimentation process the most efficient solid–liquid separation method.^6−11^ Solid–liquid separation is a common practice for various reasons, including dewatering the solid concentrates, removing solid particles from recirculation water in processing plants, cleaning liquids for environmental concerns, etc. However, in the sedimentation process, the settling time is longer as the particle size decreases. Colloidal particles can even retain a dispersed state affected by surface phenomena instead of the gravity effects associated with their size.^12^

The electrokinetic potential, or zeta potential (ζ) (expressed in ± mV), significantly impacts the solid–liquid separation processes. The magnitude of the ζ-potential changes with many factors, including the media’s pH and mineral properties, and controls the movement of colloidal particles in an aqueous medium. High ζ-potential values (−25 mV ≥ ζ ≥ + 25 mV) indicate stability and mutual repulsion among the particles, so they stay dispersed in the aqueous medium due to their meager settling rates. On the other hand, at the isoelectric point, pH_iep_, the electrical double layer (EDL) disappears and agglomeration occurs naturally.^7,12,13^ Moreover, destabilization of the dispersed mineral particles makes the mineral particles get close enough to each other and come together with the effect of London–van der Waals gravitational forces so that their masses increase. This results in an increasing settling rate of the mineral particles and can be promoted using coagulants and natural/synthetic flocculants.^12,14−16^

Freshwater supply is a significant difficulty for water-consuming industries.^17,18^ Considering that the world’s freshwater resources are scarce and decreasing day by day, the importance of water leads to wastewater being efficiently used. Repeated water use reduces the need for freshwater so that water-consuming industries can continue their production without interruption. As with other industrial processes using water, considering the sectorial size and water usage amounts, solid–liquid separation in natural stone-cutting plants is essential to get recycle clean water from wastewater effluent.^19^

According to the detailed literature review, the majority of the studies on removing turbidity from natural stone-cutting/processing plant wastewaters have been done on wastewaters that contain single mineral fines (marble, travertine, etc.). So, the researchers focus on a size-enlargement treatment to increase the settling rate of the mineral fines by using either a coagulant or a flocculant. In these experimental studies, including our previous study, it was concluded that turbidity removal efficiency varied mostly with pH, charge, and charge density of the flocculant, particle type/size in the effluent, natural stone type, the solid ratio of the slurries, etc. It was also concluded that the sign and the magnitudes of the charges of solid particles play a crucial role in the efficiency of the coagulation/flocculation processes.^8,9,15,20−22^ As in various wastewater processes in different industrial applications, an efficient and fast turbidity removal process from natural stone wastewater plays a critical role in allowing production to continue without interruption and minimizing the use of freshwater. From this point of view, this study aimed at obtaining accelerated and efficient turbidity removal from complex natural stone sizing/processing plant wastewater containing many different mineral fines such as silicates, iron-bearing minerals, etc. For this purpose, turbidity removal was achieved by facilitating solid–liquid separation comparatively by using single and binary mixtures of coagulants and flocculants with different charging mechanisms under neutral pH conditions. With the help of the findings attained, understanding the interaction mechanism between mineral particles and coagulants/flocculants, particularly near pH_iep_ values, was targeted to contribute to applying appropriate and more efficient turbidity removal processes.

## 2 Materials and Methods

### 2.1 Wastewater of the Natural Stone Processing Plant

The natural stone processing plant wastewater sample was obtained from a wastewater settling pond near Isparta, Turkey. The plant’s water usage varies between 60 and 90 m^3^/day, depending on shift/day, season, and daily production. This amount is met daily by approximately 2 m^3^ of groundwater, and the rest is met by the recirculation of the wastewater after treatment. The sample used throughout the study was obtained in 5 L sealed pet bottles. The calculated pulp density of the sample was found to be 1.25 ± 0.15% solid by weight. As the mineral fines settled, and the homogeneity of the wastewater sample changed with time, the pet bottles were shaken at least 5 min before use, and immediately after the shaking process, the homogenized sample was transferred to a 500 mL graduated cylinder for the tests.

**Figure 1:**
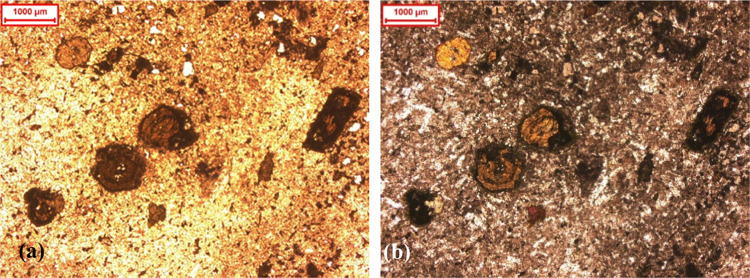
Photomicrograph of the sample in (a) parallel and (b) crossed Nicol.

X-ray fluorescence (XRF) and microscopic analyses revealed characterizations of the mineral particles in the wastewater. The results of the chemical analysis are given in Table 1, and the images of a microscopic examination done by a Leica DMLP research polarizing microscope are shown in Figure 1. According to the analyses done, it mainly contains (45%) nepheline as a primary mineral, followed by orthoclase (25%), oligoclase (15%), and amphibole (8%). The sample also contains 5% magnetite, ilmenite, and 2% pyroxene. The rest were formed by biotite, illite, and kaolin. The rock exhibits two different particle sizes and has a very fine crystalline matrix with a porphyrin characteristic because of coarse crystalline feldspathoid and amphibole minerals.

**Table 1:** Chemical Analyses of Mineral Fines in Wastewater

chemical composition	%
SiO_2_	58.14
Al_2_O_3_	18.38
Na_2_O + K_2_O	12.34
MgO + CaO	4.77
Fe_2_O_3_	4.54
others	1.83

### 2.2 Coagulants and Flocculants

The coagulants (Fe_2_SO_4_ and FeCl_3_ (anhydrous form)) used in the experimental studies were obtained from Merck. The anionic and nonionic flocculants were obtained from Cytec Inc., and the cationic flocculant was obtained from ECS Chemicals.

Before the experimental analyses, the stock solutions of each coagulant (1 g/100 mL) and flocculant (0.1 mg/100 mL) were diluted freshly based on the needs. The wastewater was stirred (500 rpm) for 5 min at the target pH and transferred to a 500 mL graduated cylinder for the tests. Following the transfer, the sample was shaken ten times before being allowed to stand still. The same procedure was applied for the turbidity removal tests with coagulants, flocculants, and their mixtures.

### 2.3 Turbidity Removal

The turbidity removal tests were done using a Hanna HI 93703 portable turbidity meter at an ambient temperature between 18 and 22 °C. The instrument has a three-point calibration system at 0–10–100 FTU (Formazine Turbidity Unit). With the device, the tests can be done between the minimum and maximum units of 0.00 and 1000 FTU with reliable results having a sensitivity of 0.5 FTU (FTU = NTU (nephelometric turbidity unit)).

## 3 Results and Discussion

### 3.1 Turbidity of Wastewater

**Figure 2:**
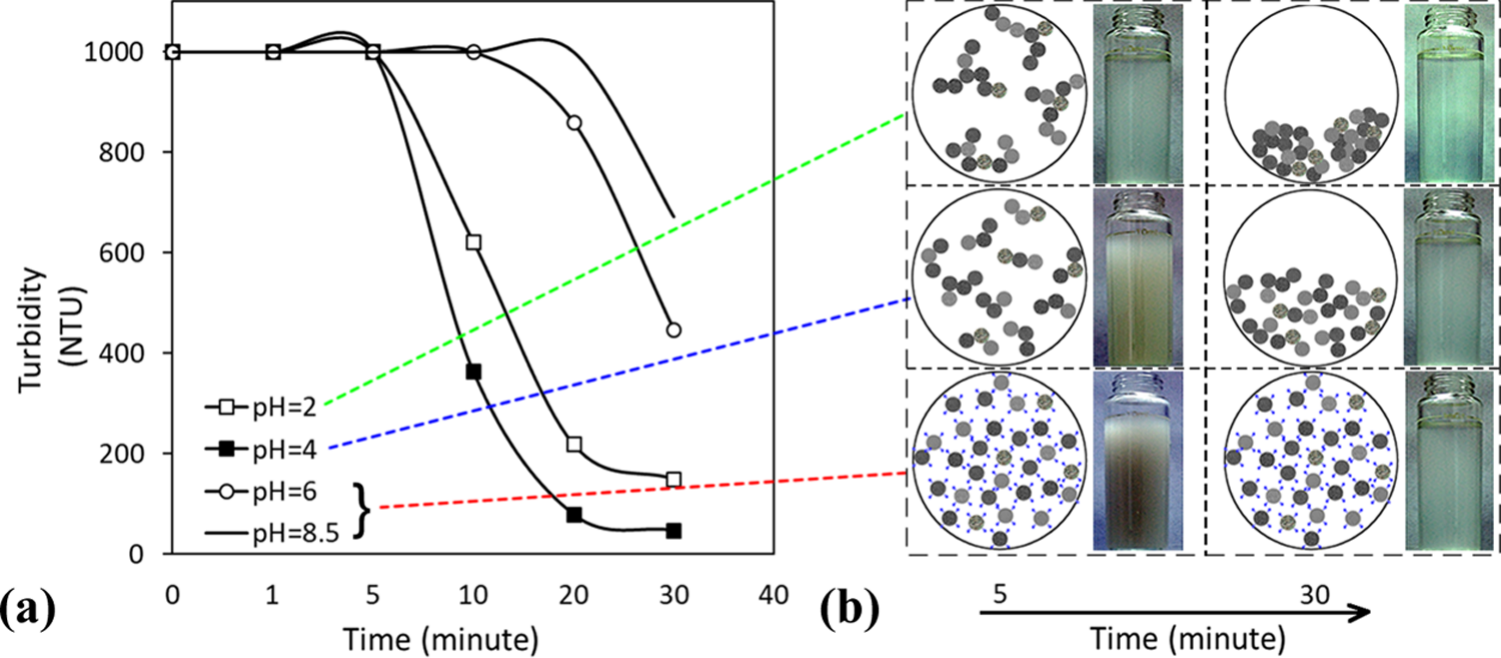
(a) Turbidity vs time profiles of the wastewater at different pH values and (b) corresponding sample cuvette images (highest (1000 NTU) to lowest turbidity (<50 NTU)).

This study aimed to investigate pH-dependent turbidity removal from wastewater containing natural stone fines using coagulants and flocculants under neutral pH conditions. The turbidity removal tests were also performed using their binary mixtures to compare the results in terms of their effectiveness in the turbidity removal process. To ensure comparability with the results obtained with the coagulants, flocculants, and their binary mixtures, pH vs turbidity value changes of the original samples were also determined between the pH range of 2 and 6 depending on the duration (0–30 min). The results of the original sample and the images of the sample cuvettes with different turbidity values are given in Figure 2a–b.

**Figure 3:**
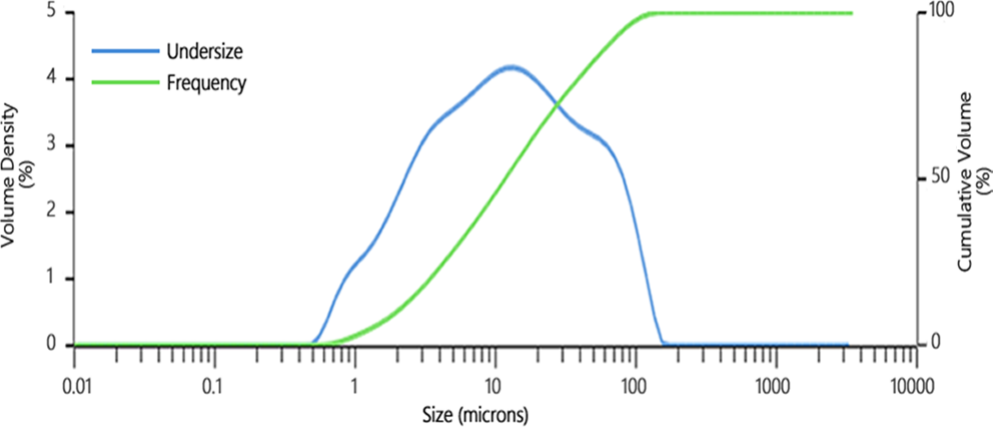
Particle size distribution of the sample.

In Figure 2a, the turbidity values of the sample change with both the pH and time. The turbidity values of the as-received sample (pH 8.5) from the start to the 20th minute remained constant at 1000 NTU, the maximum detectable turbidity value by the instrument, and decreased slightly to about 670 NTU after 30 min. Similarly, at the near-neutral pH value of 6, the turbidity values were constant at 1000 NTU in the first 10 min and then decreased gradually to about 860 NTU after 20 min and to less than 450 NTU after 30 min. The particle size distribution of the plant wastewater (determined by a Malvern Mastersizer 3000) given in Figure 3 shows that 80% passing size (*d*_80_) of mineral particles in the wastewater was calculated as 0.040 mm with a maximum size of 3.2 mm (<%0.01 by volume). Considering this value, the results clearly showed that the influence of gravitational effects related to the mass of the mineral particles became smaller. Thus, the similarly charged mineral particles repelled each other, dispersed in the wastewater, and stayed suspended, causing maximum turbidity values of 1000 NTU (pH 6 and pH 8.5) (Figure 2b). On the other hand, with the decrease in pH, the stability of the mineral particles dropped rapidly. This dramatically promoted particles to agglomerate, which resulted in turbidity values lower than those obtained over near-neutral pH values (Figure 2a). As a result, the turbidity values obtained at pH 4 were the lowest. The initial turbidity value of 1000 NTU dropped rapidly to less than 365 NTU after 10 min, 80 NTU after 20 min, and less than 50 NTU after 30 min. Regarding pH 2, the turbidity values were about 620 NTU, 220 NTU, and 150 NTU, after 10, 20, and 30 min, respectively.

### 3.2 Turbidity Removal with Coagulants

Two forces, gravity and drag, affect the settling velocity of the particles in an aquatic environment significantly. The main factors affecting gravitational force are the particle size and density. The rate of settling increases with increasing particle size and/or density. On the other hand, drag force acts in the opposite direction to the particle’s motion. It grows with the settling velocity of the mineral particles until the particles reach the terminal velocity. The terminal velocity most notably depends upon particle characteristics like size, shape, and density and aqueous environment characteristics like viscosity and density.^12^

The time required for particles in mm size to settle in 100 mm is less than a second, while the settling time for particles with the same density, for example, in colloidal size (<10^–5^ mm), may take years. This is primarily due to the surface properties of the mineral particles. With the decreasing particle size, the surface properties of the mineral particles become more effective in their movement in the solution. As given in the Section 2 of the study, the wastewater mainly contains silicate minerals, and most of the silicates, including those from our previous studies, show negative ζ-potential values over the entire pH range.^23−26^ So, depending on the magnitude of the ζ-potential values, mineral particles with the same sign repel each other in every direction and suspend in the solution. Their tendency to remain suspended decreases with decreasing ζ-potential magnitude.^27^ They become unstable when they have ζ-potential values between ±15mV, and at pH_iep_, where the electrical double layer disappears, mineral particles aggregate.

#### 3.2.1 Effect of the Coagulants on Turbidity

The thickness of EDL, so the interparticle repulsion, is strongly affected by the ionic strength of the solution. The compression of the electrical double layer is related to the amount of indifferent electrolyte and the valence of counterions (oppositely charged ions). The higher the concentration of the indifferent electrolyte and the counterion valence, the less effective the interparticle repulsion,^27^ so the more influential the coagulation. In addition to their high efficiency and availability, ferrous and ferric coagulants, having enhanced adsorption characteristics, are the most common. Regarding this, it was aimed to determine the effects of coagulants, FeSO_4_ and FeCl_3_, with varying valences of counterions (Fe^2+^, Fe^3+^, Cl^1–^, SO_4_^2–^) on turbidity removal under neutral pH conditions (20–80 mg/L).

**Figure 4:**
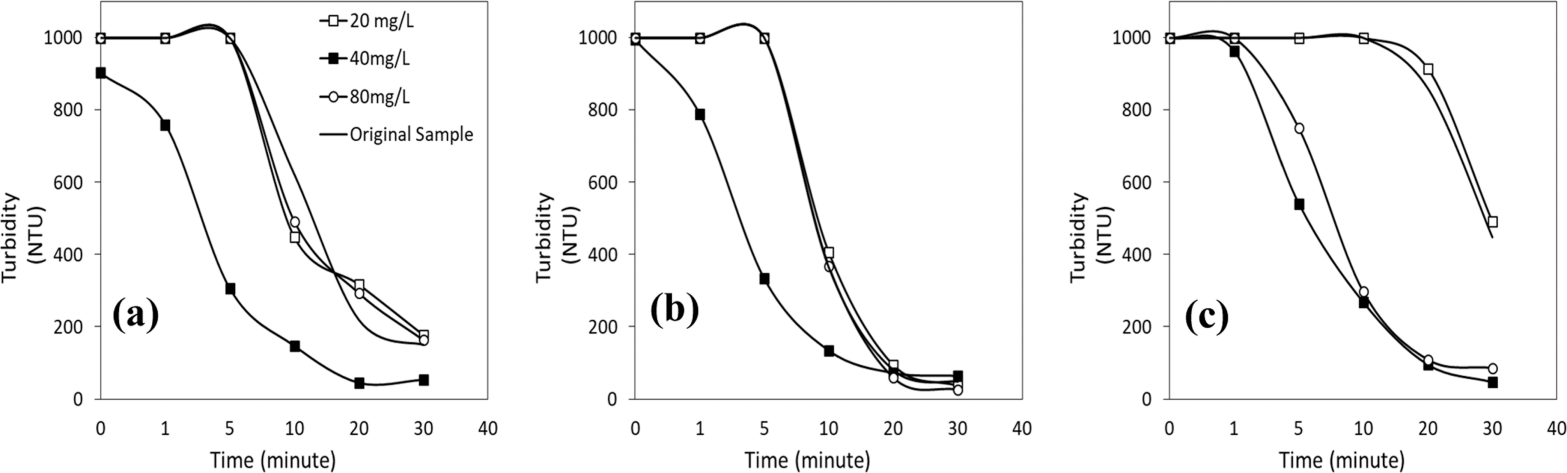
Turbidity vs time profiles of wastewater with FeSO_4_ at (a) pH 2, (b) pH 4, and (c) pH 6.

**Figure 5:**
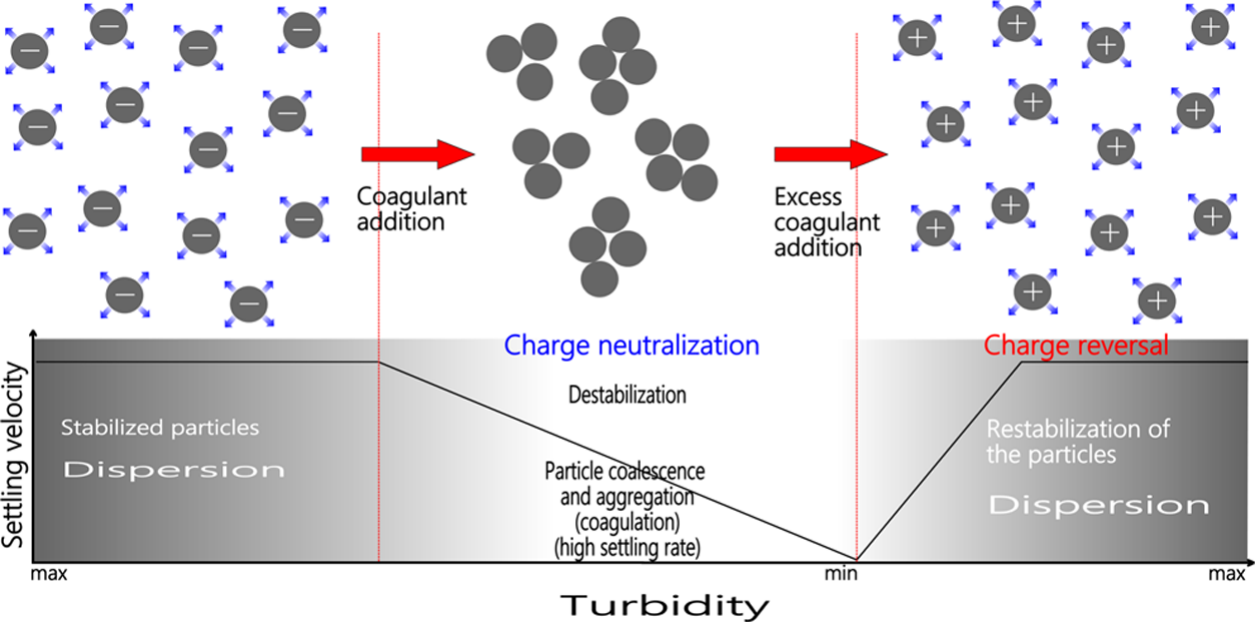
Effect of the co-addition on particle coalescence.

The results given in Figure 4a–c show that the turbidity removal with the addition of FeSO_4_ varied with the pH, concentration, and time. With 20 mg/L FeSO_4_ at each tested pH level, the turbidity values matched those obtained with the original sample (at the same pH values without coagulant). In the first 5 min of the process, the initial and peak turbidity levels stayed at 1000 NTU. After 30 min, the turbidity values steadily fell to about 160 NTU with an 84% turbidity removal effectiveness. This was due to the insufficient FeSO_4_ concentration. With increasing FeSO_4_ concentration, lower turbidity values were obtained with 40 mg/L FeSO_4_, notably at pH 2 and 4. At pH 2, the initial turbidity (903 NTU) rapidly decreased after 5 min to 306 NTU with about 66% turbidity removal efficiency and, after 30 min, subsequently to 60 NTU with about 93% turbidity removal efficiency. Turbidity values of ≤100 NTU were obtained after 20 min of coagulation process with all of the FeSO_4_ concentrations tested. Figure 5 shows that when a coagulant is added to plant wastewater, the coagulant species move toward the oppositely charged mineral surfaces in the wastewater to neutralize and destabilize them. However, using a higher coagulant concentration than needed restabilizes the mineral particles with the opposite charge, which results in higher turbidity values.^28^ Because of this, the turbidity values at pH 2 and 4 with 80 mg/L FeSO_4_ were unexpected and extremely close to those found with the original sample. The ζ-potential values of the mineral particles in the wastewater became more negative with increasing pH. Therefore, at pH 6, the effectiveness of Fe^2+^ ions increased, resulting in the turbidity values that were much lower than those of the original sample with increasing FeSO_4_ concentration.

**Figure 6:**
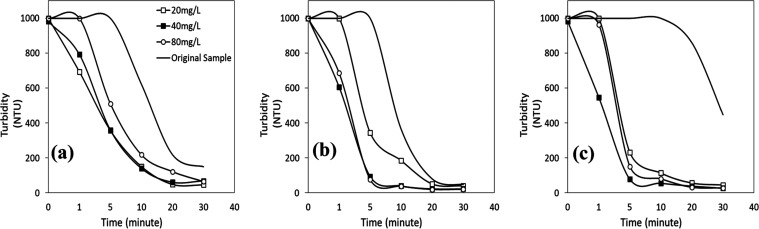
Turbidity vs time profiles of plant wastewater with FeCl_3_ at (a) pH 2, (b) pH 4, and (c) pH 6.

Because the higher-valence counterions have more potent effects on the electrical double-layer contraction, easier coagulation occurs with decreasing critical coagulation concentration (CCC) due to the increasing valence of counterions.^29^ Therefore, adding FeCl_3_ to wastewater led to turbidity values much lower than those obtained with FeSO_4_ at any pH level with any coagulant concentration tested (Figure 6a–c). As the stability of most particles in the wastewater increased with increasing pH (>pH 2), the effectiveness of FeCl_3_ increased. This resulted in less than 100 NTU turbidity values after 5 min of coagulation at certain FeCl_3_ concentrations (>90% turbidity removal efficiency) and the lowest turbidity values of less than 20 NTU after 30 min (>97%).

### 3.3 Turbidity Removal with Flocculants

Most flocculants, called polyelectrolytes, are derived from polyacrylamide (PAM), which has a nonionizing structure. They can have anionic, cationic, and nonionic structural groups. Their effectiveness varies depending on their ionization state, flocculant concentration, wastewater pH, mineral surface properties, other ions in the solution, etc.^30,31^ So, this part of the study aimed to examine the effects of anionic, cationic, and nonionic flocculants on removing turbidity from wastewater. The turbidity tests were applied using two different flocculant concentrations of 0.25 and 0.50 mg/L between 0 and 30 min at three acidic pH values. The results obtained in the presence of the flocculants are given together in each figure with the results of the original sample (without flocculant at the same pH values) to make the results more comparable.

#### 3.3.1 Turbidity Removal with an Anionic Flocculant

As given in the Section 2, the natural stone processing wastewater mainly contains silicate minerals with pH_iep_ values below pH 2. That is, most of the particles in the wastewater were negatively charged at all of the pH values tested. The wastewater also contains iron–titanium-bearing minerals with pH_iep_ values of 4–5.^32−34^ That means these minerals have positive ζ-potentials below pH_iep_ and negative ζ-potentials above pH_iep_. Therefore, when the anionic flocculant is added to the wastewater in the presence of electrostatic forces that are dominant, it is expected to be attracted by the oppositely charged mineral surfaces and repelled away by the similarly charged mineral surfaces. Lower turbidity values are obtained in the former case, while high turbidity values are obtained in the latter case.^15,35^

**Figure 7:**
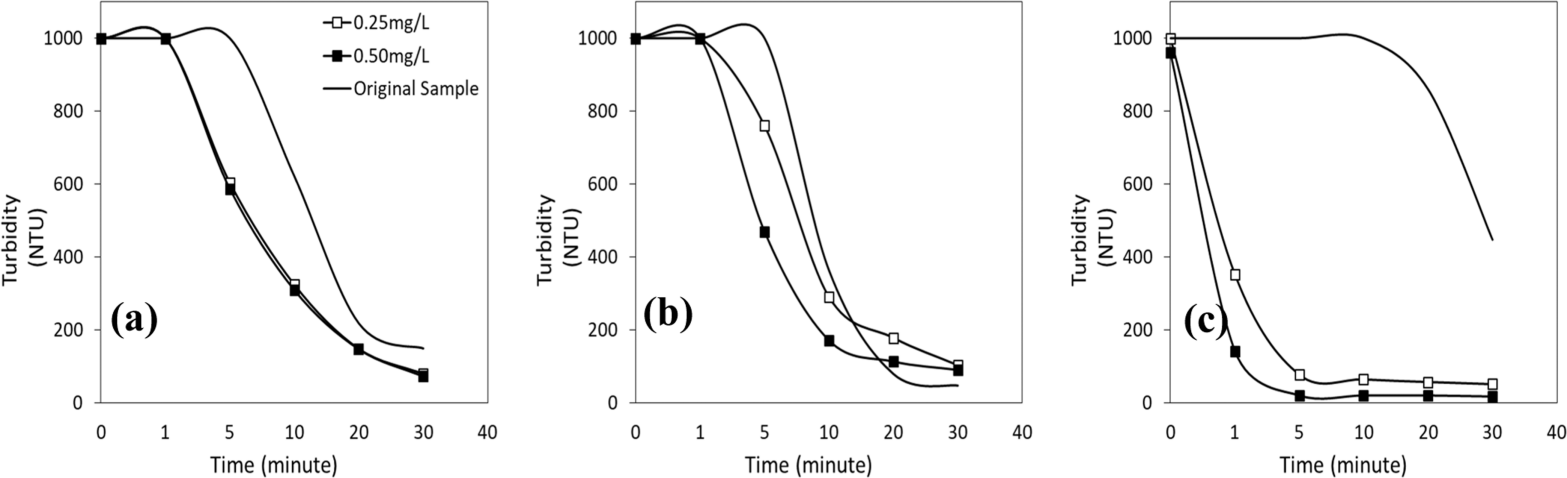
Turbidity vs time profiles of wastewater with an anionic flocculant at (a) pH 2, (b) pH 4, and (c) pH 6.

In Figure 7a–c, the results show that the effect of the anionic flocculant on the removal of turbidity at pH 2 and 4 was limited for all flocculant concentrations tested. With both flocculant concentrations, the turbidity of 1000 NTU gradually dropped to about 590 NTU after 5 min and to about 75 NTU after 30 min. A smaller percentage of the minerals in the wastewater exhibited positive ζ-potential values at pH 2, and some were destabilized since the pH was so close to pH_iep_. As a result, the larger flocs formed by the flocculant species adhering to the oppositely charged mineral particles reduced the turbidity (Figure 7a). At pH 6 (Figure 7c), given that the majority of the wastewater’s mineral particles had charges similar to those of the flocculant, higher turbidity values were anticipated as a result of electrostatic repulsion between the anionic flocculant and the mineral particles. On the other hand, the anionic flocculant yielded the best removal efficiency for turbidity at pH 6. The initial turbidity of 1000 NTU immediately decreased to 353 NTU with a flocculant concentration of 0.25 mg/L and 960 NTU to 150 NTU with a flocculant concentration of 0.50 mg/L in the first minute. After 5 min, the best turbidity removal efficiency values were >92 and >97% for flocculant concentrations of 0.25 and 0.50 mg/L, respectively. That might be due to the activation of negatively charged mineral particles with the effect of positively charged high-valence counterions (Ca^2+^, Mg^2+^, etc.) in the wastewater.^15,36^ As a result, the oppositely charged ions present in the wastewater led to the possibility of positive sites on mineral surfaces for the adsorption of the anionic flocculant via a cation-bridging effect.^12,37,38^

#### 3.3.2 Turbidity Removal with Cationic Flocculant

**Figure 8:**
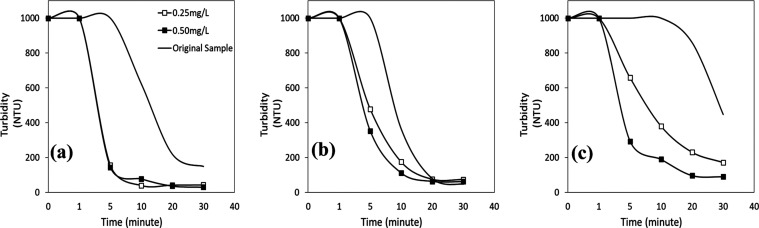
Turbidity vs time profiles of wastewater with a cationic flocculant at (a) pH 2, (b) pH 4, and (c) pH 6.

Cationic flocculants are polymers with positively charged functional groups. They are used to flocculate and settle negatively charged particles. Figure 8a–c demonstrates that the cationic flocculant was most effective at pH 2 and 4. The initial turbidity value of 1000 NTU reduced rapidly to 150 NTU after 5 min with a turbidity removal efficiency of approximately 85%, as per the results for both flocculant concentrations at pH 2 (Figure 8a). After 10 min of the flocculation process, the turbidity removal efficacy for the given pH was around 95%.

At pH 4 (Figure 8b), the majority of the silicate minerals in the wastewater possessed moderately strong negative ζ-potentials, which prompted the oppositely charged cationic flocculant to interact. The iron–titanium-bearing minerals in the wastewater did not show net positive or negative surface properties and remained destabilized at pH conditions close to pH_iep_. When these mineral particles became unstable in the solution, the attractive interactions between oppositely charged flocculant species and mineral particles diminished. As a result, the turbidity removal efficiencies were similar for each flocculant concentration and ranged from 50 to 60% after 5 min of flocculation to nearly 90% after 20 min.

The stability of the mineral particles in the wastewater increased with pH, producing substantially higher turbidity values with the original sample (the highest turbidity values at all pH values tested remained at 1000 NTU for 10 min) than with the cationic flocculant. At pH 6 (Figure 8c), the turbidity dropped slowly to 660 NTU and 175 NTU, after 5 and 30 min, respectively, with the flocculant concentration of 0.25 mg/L. The initial turbidity value significantly dropped in the flocculant concentration of 0.50 mg/L, from 1000 NTU to 300 NTU after only 5 min and 100 NTU after 30. Even though most of the wastewater’s minerals exhibited strong negative ζ-potential values at pH 6, the electrostatic attraction might be the leading cause of the decreased turbidity values. However, these results confirmed that, as explained in the turbidity removal with an anionic flocculant (Section 3.3.1), the interaction of the minerals with the cationic flocculant was avoided by high-valence counterions in the wastewater that led to charge reversal on the surfaces of the minerals. As a result, against predictions, both cationic flocculant concentrations produced substantially higher turbidity values than those at pH 2 and 4, respectively.

#### 3.3.3 Turbidity Removal with a Nonionic Flocculant

**Figure 9:**
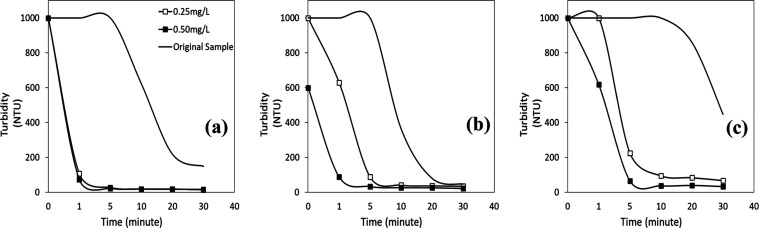
Turbidity vs time profiles of wastewater with a nonionic flocculant at (a) pH 2, (b) pH 4, and (c) pH 6.

Unlike ionic flocculants (anionic or cationic flocculants), nonionic flocculants do not carry a net positive or negative charge. Their adsorption mechanism differs from that of ionic flocculants and does not rely on electrostatic interactions with charged particles or surfaces. In Figure 9a–c, the outcomes demonstrate that pH, flocculant concentration, and time all affected how well nonionic flocculants removed turbidity from wastewater. At pH 2 (Figure 9a), the flocculation process for both concentrations was rapid and resulted in less than 20 NTU with a turbidity removal efficiency of 98% in the first minute. The flocculant’s effect on turbidity reduction varied with concentration as pH increased (Figure 9b). At pH 4, the nonionic flocculant concentration of 0.25 mg/L caused initial turbidity of 1000 NTU to rapidly reduce to 630 NTU within the first minute and roughly 90 NTU after 5 min of flocculation. When the flocculant concentration was 0.50 mg/L, the starting turbidity of 600 NTU rapidly decreased to less than 90 NTU in the first minute. After 30 min of flocculation, it reached a level of roughly 20 NTU. The results also demonstrated that the nonionic flocculant interactions with mineral particles reduced as particle stability increased, leading to significantly higher turbidity values for both flocculant concentrations. The initial turbidity value of 1000 NTU for the same flocculant concentration at pH 6 (Figure 9c) steadily fell to about 70 NTU after 5 min of flocculation. After 5 min, the results were less favorable than those with the flocculant concentration of 0.25 mg/L (about 225 NTU).

### 3.4 Turbidity Removal with Binary Coagulant/Flocculant Mixtures

When turbidity is removed from wastewater, combining coagulants and flocculants and using them as binary mixtures can occasionally be more effective instead of using them separately. With the destabilization of the mineral particles by neutralizing ζ-potential using coagulants, they can get closer to each other and are held together by the help of weak van der Waals forces. Therefore, improved turbidity removal efficiencies can boost floc stability while reducing coagulant/flocculant usage using binary coagulant/flocculant mixes.

**Figure 10:**
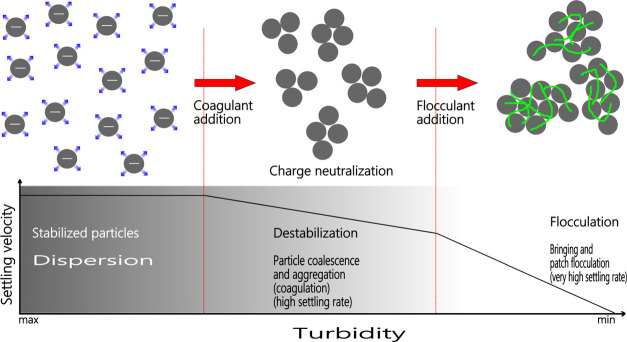
Schematic representation of the effect of coagulant/flocculant mixtures on turbidity.

**Figure 11:**
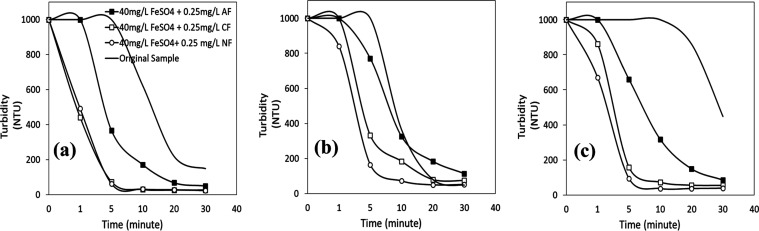
Turbidity vs time profiles of wastewater with 40 mg/L FeSO_4_ + 0.25 mg/L flocculant mixtures at (a) pH 2, (b) pH 4, and (c) pH 6.

According to the results obtained with their single use, it was demonstrated that the mineral particles’ ζ-potential values affect how flocculants adhere to the mineral particles and their capacity to reduce turbidity values. Despite having comparable charges, mineral particles and flocculants occasionally achieved significant turbidity removal efficiency^39^ under specific pH situations, such as when the mineral particles’ pH_iep_ values were reached. Therefore, in this part of the study, it was aimed to ascertain how binary coagulant/flocculant mixes affected the reduction of turbidity from wastewater depending on the previously selected coagulant (FeSO_4_, FeCl_3_), and flocculant (anionic (AF), cationic (CF), and nonionic (NF) flocculants) concentrations at pH 2–6. For the turbidity tests using coagulant/flocculant mixes, the wastewater was conditioned with coagulant for 5 minutes and agitated for an additional 5 min after adding the flocculant at the desired pH level. The results in Figures 10 and 11a–c show that the effectiveness of coagulant/flocculant mixes on turbidity removal from wastewater varied with pH, flocculant type, and duration of the process.

In Figure 11a, the turbidity removal efficiencies obtained with mixtures of cationic and nonionic flocculants at pH 2 were similar and about 94% at 5 min after the process started. When the results obtained with the single use of FeSO_4_ (40 mg/L) at pH 2 (Figure 4a) were taken into account, the decreasing stability of the mineral particles in the wastewater promoted the adsorption of cationic and nonionic flocculants, which resulted in much lower turbidity values than in the case of original sample (at the same pH values without a coagulant or flocculant or their binary mixes). So, the lowest turbidity values were obtained with the mixture prepared with cationic and nonionic flocculants at pH 2. The impact of pH, near the pH_iep_ value of several minerals in the wastewater, may also have contributed to the decreased turbidity results. With the coagulant/anionic flocculant mixture, the turbidity values were almost the same as the results obtained with the original sample at pH 2 and 4 (Figure 11a–b), confirming no/low interaction of the anionic flocculant with the mineral particles after the coagulant addition.

The increase in pH increased the ζ-potential magnitude of the minerals in the wastewater with no additives (pH 6). So, similarly charged mineral particles repelled each other more strongly, displaying high turbidity levels that remained steady for 10 min at 1000 NTU (Figure 11c). The results also demonstrated that the oppositely charged Fe^2+^ ions reduced the magnitude of the mineral particles’ ζ-potential values after adding 40 mg/L FeSO_4_ to the wastewater. This led to lower turbidity values than those obtained with the original sample. This promoted the turbidity removal efficiencies for cationic and nonionic flocculants, resulting in turbidity removal efficiencies >85 and >90% after 5 min and about 95% after 30 min, respectively. Higher turbidity values than in the case of the single usage of the anionic flocculant resulted from the mineral particles’ surfaces not being adequately neutralized, which led to an electrostatic repulsion with the anionic flocculant species.

Due to the compression of the electrical double layer, the interparticle repulsion can be reduced by increasing the ionic strength of the wastewater. As the concentration of ions in the wastewater increases, the ionic strength of the wastewater increases, causing a transition of the mineral particles from stability to destabilization. However, the concentration of the ions in the wastewater is essential and can be effective over a narrow range of electrolyte concentrations. While the concentration of the ions is sufficient to destabilize the minerals in the wastewater, increasing the concentration a bit could result in the ineffectiveness of the ions, promoting charge reversal.^28^

**Figure 12:**
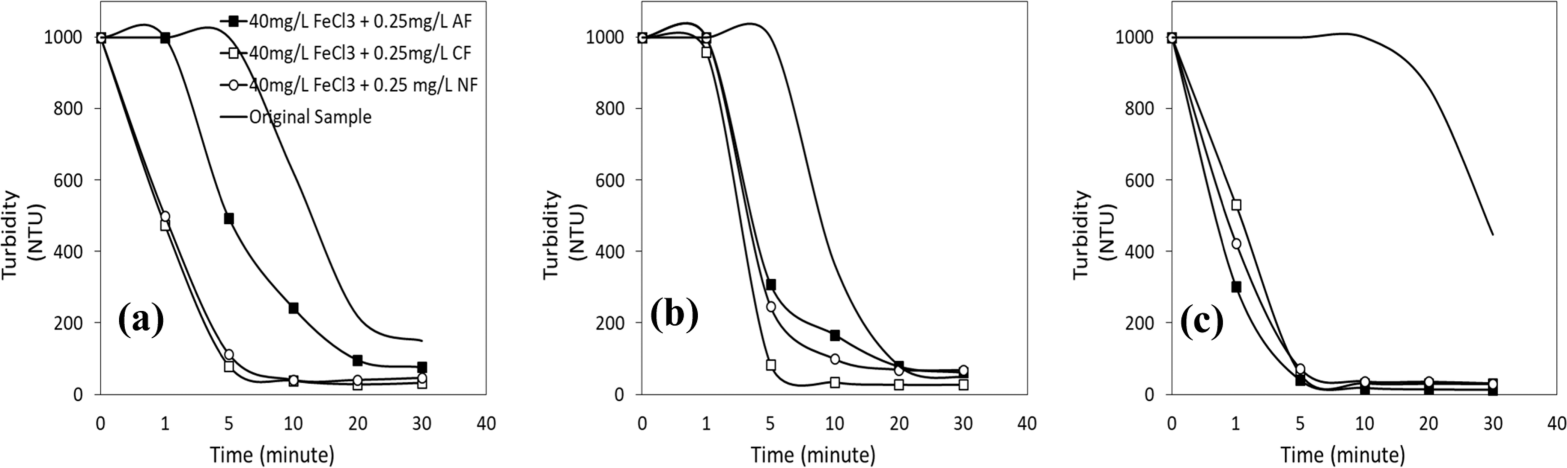
Turbidity vs time profiles of wastewater with 40 mg/L FeCl_3_ + 0.25 mg/L flocculant mixtures at (a) pH 2, (b) pH 4, and (c) pH 6.

The valence of the counterions significantly impacts the electrolyte concentration but is independent of the mineral content in the effluent. In this part of the study, the impact of coagulant/flocculant mixes using FeCl_3_ is examined from this perspective. According to the results in Figure 12a–c, given the same concentrations, the results obtained at pH 2 were almost similar to those obtained with FeSO_4_ and compared to those using FeSO_4_, and the efficacy of the binary coagulant/flocculant mixes of anionic and cationic flocculants increased with increasing coagulant valence at pH 4 and 6. At pH 4 (Figure 12b), the turbidity value of 1000 NTU after 5 min decreased to about 300 NTU with the anionic flocculant and to >100 NTU with the cationic flocculant, with turbidity removal efficiencies of about 70 and 90%, respectively. At pH 6 (Figure 12c), all turbidity removal efficiency lines with each flocculant mixture were almost similar, with turbidity removal efficiency values over 90% after 5 min. The turbidity values obtained at pH 6 were much lower than those obtained with each coagulant’s and each flocculant’s single use. The results confirmed that using a higher-valence cation (Fe^3+^) compressed the mineral particles’ electrical double layer, promoting the adsorption of flocculants, especially anionic and cationic flocculants, on their surfaces.

At pH 2 and 4, which are the closest pH values to the pH_iep_ of some minerals, the stability of those minerals in the wastewater was low, so some particles remained stable, some underwent charge reversal, and some destabilized compared to those at pH 6 (Figure 2a). Therefore, the results also showed that due to the pH-dependent hydrolysis reaction of the nonionic flocculant,^40,41^ electrostatic interaction between the nonionic flocculant and mineral particles could be one of the possible mechanisms for the higher turbidity values compared to those with a single use of the nonionic flocculant. The results at pH 6 also showed that the low turbidity values obtained could be the result of nonelectrical interactions as well as hydrogen bonding that occurred between mineral particles and each flocculant, even if the majority of minerals in the wastewater had negative ζ-potential values or the use of a coagulant affected their charge reversal.

## 4 Conclusions

In this study, the effects of three flocculants with different charging mechanisms (anionic, cationic, and nonionic) and two different coagulants with varying valences of counterion (FeSO_4_, FeCl_3_) on the turbidity removal from natural stone fines-containing wastewater were compared depending on concentration, time, and pH. Another goal of the study was to determine how binary coagulant/flocculant mixtures affected the reduction of turbidity at particular concentrations and pH levels.

With the same concentrations, the coagulant’s efficiency on EDL compression rose as the counterion valence did, leading to much lower turbidity values and higher turbidity removal efficiencies. According to the findings, 160 mg/L FeSO_4_ (about 97%) at pH 4 and 80 mg/L FeCl_3_ (about 99%) at the same pH provided the maximum turbidity reduction effectiveness.

The turbidity removal efficiencies with flocculants varied with concentration and pH. The best turbidity removal efficiencies were obtained with a nonionic flocculant at pH 2, where most of the minerals in the wastewater were unstable because the pH was close to their pH_iep_ values. Under the given conditions, the turbidity of the wastewater decreased rapidly from the initial turbidity value of 1000 NTU to less than 75 NTU in the first minute of the process. The effectiveness of the cationic flocculant was higher at pH, where some of the minerals in the wastewater had negative ζ-potential values. In the case of an anionic flocculant, even though most of the minerals were stable and negatively charged, the lower turbidity values were obtained with about 98% turbidity removal efficiency after 5 min at pH 6 because of the charge reversal effect of the high-valence counterions available in the wastewater.

The results also showed that in comparison to using the coagulants and flocculants separately at higher concentrations binary coagulant/flocculant mixtures promoted the aggregation of mineral particles and produced much lower turbidity values with the use of lower coagulant/flocculant concentrations.

The maximum turbidity removal efficiency with binary coagulant/flocculant mixes was >95% at pH 2 with FeSO_4_ and pH 6 with FeCl_3_, respectively, after 10 min. With time, they became more efficient in removing turbidity, reaching turbidity removal efficiencies of >98% under specific pH conditions.
